# ﻿Morphological and phylogenetic analyses reveal four novel species of *Distoseptispora* (Distoseptisporaceae, Distoseptisporales) from southern China

**DOI:** 10.3897/mycokeys.113.137082

**Published:** 2025-01-27

**Authors:** Ming-Gen Liao, Xing-Xing Luo, Ya-Fen Hu, Rafael F. Castañeda-Ruíz, Zhao-Huan Xu, Jian Ma

**Affiliations:** 1 College of Agronomy, Jiangxi Agricultural University, Nanchang, Jiangxi 330045, China; 2 Instituto de Investigaciones de Sanidad Vegetal, Calle 110 No. 514 e/5ta B y 5ta F, Playa, La Habana 11600, Cuba; 3 Jiangxi Key Laboratory for Excavation and Utilization of Agricultural Microorganisms, Jiangxi Agricultural University, Nanchang, Jiangxi 330045, China

**Keywords:** Asexual fungi, molecular phylogeny, new species, Sordariomycetes, taxonomy

## Abstract

Saprobic hyphomycetes with high density and species diversity were observed on plant debris. During our mycological surveys in southern China, numerous strains were isolated from dead branches of unidentified plants in the forest of Jiangxi and Fujian provinces, China. Phylogenetic analyses of ITS, LSU, *RPB2*, and *TEF1* sequence data using maximum-likelihood (ML) and Bayesian inference (BI) methods revealed the systematic placement of several *Sporidesmium*-like species within *Distoseptispora*. Based on morphological characteristics and molecular evidence, four new species of *Distoseptispora*, namely *D.fujianensis*, *D.ganzhouensis*, *D.nanpingensis* and *D.subtropica*, are introduced, and two known species, *D.clematidis* and *D.yunjushanensis*, are reported. This study enhances our knowledge of the species diversity of *Distoseptispora* in southern China.

## ﻿Introduction

The genus *Distoseptispora* K.D. Hyde, McKenzie & Maharachch., typified by *D.fluminicola* McKenzie, Hong Y. Su, Z.L. Luo & K.D. Hyde, was introduced by Su et al. (2016) based on molecular data and is mainly characterized by acrogenous, solitary, distoseptate conidia seceding schizolytically from monoblastic, integrated, terminal, determinate conidiogenous cells on macronematous, unbranched, septate conidiophores. Subsequently, the generic concept has been expanded by adding species that produce euseptate or muriform conidia (Xia et al. 2017; Yang et al. 2018) and polyblastic conidiogenous cells (Hyde et al. 2019). To date, 84 epithets of *Distoseptispora* are listed in Index Fungorum (Index Fungorum 2024), with all species identified based on morphological and phylogenetic analyses. However, *D.submersa* Z.L. Luo, K.D. Hyde & H.Y. Su (Luo et al. 2018), a phylogenetically close species, showed larger conidiophores (55–73 × 7–9 μm vs. up to 40 × 4–6 μm) than the holotype of *D.tectonae* Doilom & K.D. Hyde (Hyde et al. 2016). Another collection of *D.tectonae* (MFLU 20–0262) also showed varied, larger conidiophore dimensions (34–95 × 5–8 μm) (Sun et al. 2020). As a result, Dong et al. (2021) synonymized *D.submersa* with *D.tectonae* based on high homology in nucleotide sequences and morphological similarity. Thus, *Distoseptispora* currently includes 83 valid taxa.

*Distoseptispora* is one of the *Sporidesmium*-like genera found in freshwater and terrestrial habitats. In recent years, the number of *Distoseptispora* species has steadily increased, primarily based on morphological and phylogenetic analyses. Recent studies have indicated that the use of ITS, LSU, *RPB2*, and *TEF1* markers provide a more accurate evaluation of their phylogenetic relationships and taxonomic placements (Hu et al. 2023; Shen et al. 2024). Most *Distoseptispora* species have been introduced based on their asexual morphs, with only two species, *D.hyalina* and *D.licualae*, described from their sexual morphs. However, the connection between sexual- and asexual morph has not yet been confirmed through cultural studies or molecular data (Yang et al. 2021; Konta et al. 2023; Liu et al. 2023).

Fungal diversity in southern China is high but remains largely unexplored. During our ongoing mycological survey of mitosporic fungi in the forest ecosystems of southwest China, several *Sporidesmium*-like taxa were isolated from dead branches of unidentified plants in Jiangxi and Fujian provinces, China. Both morphological data and multi-loci (ITS, LSU, *RPB2*, and *TEF1*) phylogenetic analyses placed six species within the genus *Distoseptispora*, including four new species, namely *D.fujianensis*, *D.ganzhouensis*, *D.nanpingensis* and *D.subtropica*, as well as two known species, viz. *D.clematidis* Phukhams., M.V. de Bult & K.D. Hyde and *D.yunjushanensis* Z.J. Zhai & D.M. Hu. Illustrations and morphological descriptions are provided, and the phylogenetic tree has been updated.

## ﻿Materials and methods

### ﻿Sample collection, fungal isolation, and morphological observation

Samples of dead branches were collected from forest ecosystems in Jiangxi and Fujian provinces, China, and placed in Ziploc™ bags for transport to the laboratory. The samples were treated following the methods described by Ma et al. (2011). The macroscopic structure of the colonies on the surface of the dead branches was examined and observed using a stereomicroscope (Motic SMZ-168, Xiamen, China) at magnifications ranging from low (7.5 ×) to high (50 ×). Fresh colonies were picked using sterile needles under 50 × magnification and placed on a slide with a drop of lactic acid–phenol solution (lactic acid, phenol, glycerin, sterile water; 1:1:2:1, respectively). The slide was then examined under an Olympus BX 53 light microscope equipped with an Olympus DP 27 digital camera (Olympus Optical Co., Ltd., Tokyo, Japan) to describe the microscopic morphological characteristics. A toothpick dipped in sterile water was used to collect conidia from the target colony on the surface of the dead branches. The conidia were placed on the surface of PDA (20% potato + 2% dextrose + 2% agar, w/v) and incubated overnight at 25 °C. Single germinated conidia were transferred to fresh PDA following the methods described by Goh (1999), and incubated at 25 °C. The morphological characteristics of the cultures including color, shape, and size, were observed and recorded. All fungal strains were stored in 10% sterilized glycerin at 4 °C for further studies. The studied specimens and cultures were deposited in the Herbarium of Jiangxi Agricultural University, Plant Pathology, Nanchang, China (HJAUP). The new species names were registered in Index Fungorum (Index Fungorum 2024).

### ﻿DNA extraction, PCR amplification, and sequencing

Fungal hyphae were scraped with sterilized toothpicks and transferred to 2 mL safe-lock tubes, then ground with liquid nitrogen. Genomic DNA was extracted using the Solarbio Fungal Genomic DNA Extraction Kit (Beijing Solarbio Science & Technology Co., Ltd., Beijing, China) according to the manufacturer’s instructions. DNA amplification was performed by polymerase chain reaction (PCR) using the loci ITS, LSU, *RPB2*, and *TEF1*. The primer pairs used for these genes were as follows: ITS: ITS5/ITS4 (White et al. 1990), LSU: 28S1–F/28S3–R (Xia et al. 2017), *RPB2*: *RPB2*–5F2 (Sung et al. 2007) /fRPB2–7cR (Liu et al. 1999), and *TEF1*: EF1–983F/EF1–2218R (Rehner and Buckley 2005; Zhao et al. 2018). The PCR reaction was carried out in a total volume of 25 µL, containing 12.5 µL of 2 × Power Taq PCR MasterMix, 9.5 µL of double-distilled water (ddH_2_O), 1 µL of DNA template, and 1 µL of each forward and reverse primer. The PCR reaction conditions for ITS, LSU, and *TEF1*, were as follows: initial denaturation at 94 °C for 3 min, followed by 35 cycles of denaturation at 94 °C for 15 s, annealing at 54 °C for 15 s, elongation at 72 °C for 30 s, with a final extension at 72 °C for 10 min, and finally held at 4 °C. The conditions for *RBP2* were similar, except annealing was performed at 59 °C, and the elongation step was extended to 2 min. The PCR products were checked on 1% agarose gel electrophoresis stained with ethidium bromide. Purification and DNA sequencing were performed at Beijing Tsingke Biotechnology Co., Ltd., Beijing, China.

### ﻿Phylogenetic analyses

The sequence datasets, comprising newly generated sequences and related sequences obtained from GenBank (Table 1), were aligned using MAFFTv.7 (Katoh and Standley 2013) on the online server (https://mafft.cbrc.jp/alignment/server/index.html, accessed on 20 April 2024), and manually optimized to improve alignment and sequence approximation. Phylogenetic analyses were carried out for each individual locus (ITS, LSU, *RPB2*, and *TEF1*), after which the four datasets were concatenated using Phylosuite software v1.2.2 (Zhang et al. 2020). The phylogenetic tree was constructed based on the concatenated ITS, LSU, *RPB2*, and *TEF1* sequence datasets using Phylosuite software v1.2.2 (Zhang et al. 2020). The concatenated and aligned datasets were analyzed separately using maximum-likelihood (ML) and Bayesian inference (BI). ML phylogenies were inferred using IQ-TREE (Nguyen et al. 2015) under an edge-linked partition model with 10,000 ultrafast bootstraps (Minh et al. 2013). The final tree was selected from suboptimal trees of each run by comparing the likelihood scores, with the SYM+I+G4 model used for ITS, TN+F+R5 for LSU and *TEF1*, and TIM3e+R3 for *RPB2*. Bayesian inference (BI) phylogenies were inferred using MrBayes 3.2.6 (Ronquist et al. 2012) under a partition model (two parallel runs, 2,000,000 generations), discarding the initial 25% of sampled data as burn-in. The best-fit model was SYM+I+G4 for ITS, and GTR+F+I+G4 for LSU, *RPB2* and *TEF1*. The ModelFinder function was used to select the best fitting partition model (Edge-linked) (Kalyaanamoorthy et al. 2017), using the BIC criterion for constructing IQ-TREE and the AlCc criterion for MrBayes. These trees were visualized using FigTree v. 1.4.4 (http://tree.bio.ed.ac.uk/software/figtree, accessed on 8 September 2024), with further editing and composition performed in Adobe Illustrator CS v. 5. The sequences generated in this study were deposited in GenBank.

**Table 1.** List of *Distoseptispora* species and GenBank accession numbers used in the phylogenetic analyses.

Taxon	Strain Number	GenBank Accession Numbers			
		ITS	LSU	*RPB2*	*TEF1*
*Acrodictysbambusicola*	CGMCC 3.18641^T^	KU999973	KX033564	—	—
*A.elaeidicola*	CGMCC 3.18642	KU999978	KX033569	—	—
*Aquapteridosporaaquatica*	MFLUCC 17–2371^T^	MW286493	MW287767	—	—
*A.fusiformis*	MFLUCC 18–1606^T^	MK828652	MK849798	—	MN194056
*A.lignicola*	MFLUCC 15–0377^T^	MZ868774	KU221018	MZ892986	MZ892980
*Bullimycesaurisporus*	AF316–1b^T^	—	JF775590	—	—
*B.communis*	AF281–5	—	JF775587	—	—
*Cancellidiumapplanatum*	CBS 337.76^T^	MH860985	MH872755	—	—
*Cancellidiumcinereum*	MFLUCC 18–0424^T^	MT370353	MT370363	MT370486	MT370488
*Distoseptisporaadscendens*	HKUCC 10820	—	DQ408561	DQ435092	—
*D.amniculi*	MFLUCC 17–2129^T^	MZ868770	MZ868761	MZ892982	—
*D.appendiculata*	MFLUCC 18–0259^T^	MN163009	MN163023	—	MN174866
*D.aqualignicola*	KUNCC 21–10729^T^	OK341186	ON400845	OP413474	OP413480
*D.aquamyces*	KUNCC 21–10732^T^	OK341187	OK341199	OP413476	OP413482
*D.aquatica*	MFLUCC 18–0646	MK828648	MK849793	—	MN194052
*D.aquisubtropica*	GZCC 22–0075^T^	ON527933	ON527941	ON533685	ON533677
*D.arecacearum*	MFLUCC 23–0212	OR354399	OR510860	OR481048	OR481045
*D.atroviridis*	GZCC 20–0511^T^	MZ868772	MZ868763	MZ892984	MZ892978
*D.atroviridis*	GZCC 19–0531	MW133915	MZ227223	—	MZ206155
*D.bambusae*	MFLUCC 20–0091^T^	MT232713	MT232718	MT232881	MT232880
*D.bambusicola*	GZCC 21–0667^T^	MZ474873	MZ474872	—	OM272845
*D.bangkokensis*	MFLUCC 18–0262^T^	MZ518205	MZ518206	—	OK067246
*D.cangshanensis*	MFLUCC 16–0970^T^	MG979754	MG979761	—	MG988419
*D.caricis*	CPC 36498^T^	MN562124	MN567632	MN556805	—
*D.chinensis*	GZCC 21–0665^T^	MZ474871	MZ474867	—	MZ501609
*D.clematidis*	MFLUCC 17–2145^T^	MT310661	MT214617	MT394721	—
***D.clematidis***	**HJAUP C1319**	**PQ211102**	**PQ211110**	**PQ303676**	**PQ303681**
*D.chishuiensis*	GZCC 23-0729^T^	PP663310	PP584670	—	PP584767
*D.crassispora*	KUMCC 21–10726^T^	OK310698	OK341196	OP413473	OP413479
*D.curvularia*	KUMCC 21–10725^T^	OK310697	OK341195	OP413472	OP413478
*D.cylindricospora*	DLUCC 1906^T^	OK491122	OK513523	—	OK524220
*D.dehongensis*	KUMCC 18–0090^T^	MK085061	MK079662	—	MK087659
*D.dipterocarpi*	MFLUCC 22–0104^T^	OP600053	OP600052	OP595140	—
*D.effusa*	GZCC 19–0532^T^	MW133916	MZ227224	—	MZ206156
*D.eleiodoxae*	MFLUCC 23–0214	OR354398	OR510859	OR481047	OR481044
*D.euseptata*	MFLUCC 20–0154^T^	MW081539	MW081544	MW151860	—
*D.euseptata*	DLUCC S2024	MW081540	MW081545	MW084996	MW084994
*D.fasciculata*	KUMCC 19–0081^T^	MW286501	MW287775	—	MW396656
*D.fluminicola*	DLUCC 0391	MG979755	MG979762	—	MG988420
*D.fluminicola*	DLUCC 0999	MG979756	MG979763	—	MG988421
***D.fujianensis***	**HJAUP C2509^T^**	**PQ211095**	**PQ211103**	**PQ303679**	**PQ303682**
***D.fujianensis***	**HJAUP C2513**	**PQ211098**	**PQ211106**	**PQ303680**	**PQ303683**
*D.fusiformis*	GZCC 20–0512^T^	MZ868773	MZ868764	MZ892985	MZ892979
***D.ganzhouensis***	**HJAUP C1090^T^**	**PQ211100**	**PQ211108**	—	**PQ303687**
*D.gasaensis*	HJAUP C2034^T^	OQ942896	OQ942891	—	OQ944455
*D.guanshanensis*	HJAUP C1063^T^	OQ942894	OQ942898	OQ944458	OQ944452
*D.guizhouensis*	GZCC 21–0666^T^	MZ474868	MZ474869	MZ501611	MZ501610
*D.guttulata*	MFLU 17–0852^T^	MF077543	MF077554	—	MF135651
*D.hainanensis*	GZCC 22-2047^T^	OR427328	OR438894	OR449119	OR449122
*D.hyalina*	MFLUCC 17–2128^T^	MZ868769	MZ868760	MZ892981	MZ892976
*D.hydei*	MFLUCC 20–0481^T^	MT734661	MT742830	MT767128	—
*D.jinghongensis*	HJAUP C2120^T^	OQ942897	OQ942893	—	OQ944456
*D.lancangjiangensis*	DLUCC 1864^T^	MW723055	MW879522	—	—
*D.lanceolatispora*	GZCC 22-2045^T^	OR427329	OR43BB95	OR449120	OR449123
*D.leonensis*	HKUCC 10822	—	DQ408566	DQ435089	—
*D.licualae*	MFLUCC 14–1163A^T^	ON650686	ON650675	—	ON734007
*D.licualae*	MFLUCC 14–1163B^T^	ON650687	ON650676	—	ON734008
*D.licualae*	MFLUCC 14–1163C^T^	ON650688	ON650677	—	—
*D.lignicola*	MFLUCC 18–0198^T^	MK828651	MK849797	—	—
*D.lignicola*	GZCC 19–0529	MW133911	MZ227219	—	MZ206152
*D.liupanshuiensis*	GZCC 23-0730^T^	PP663309	PP584669	—	PP584766
*D.longispora*	HFJAU 0705^T^	MH555359	MH555357	—	—
*D.longnanensis*	HJAUP C1040^T^	OQ942887	OQ942886	—	OQ944451
*D.martinii*	CGMCC 3.18651^T^	KU999975	KX033566	—	—
*D.meilingensis*	JAUCC 4727^T^	OK562390	OK562396	—	OK562408
*D.meilingensis*	JAUCC 4728^T^	OK562391	OK562397	—	OK562409
*D.menghaiensis*	HJAUP C2045^T^	OQ942890	OQ942900	—	—
*D.menglunensis*	HJAUP C2170^T^	OQ942899	OQ942888	OQ944461	OQ944457
*D.mengsongensis*	HJAUP C2126^T^	OP787876	OP787874	—	OP961937
*D.multiseptata*	MFLUCC 15–0609^T^	KX710145	KX710140	—	MF135659
*D.multiseptata*	MFLU 17–0856	MF077544	MF077555	MF135644	MF135652
*D.nabanheensis*	HJAUP C2003^T^	OP787873	OP787877	—	OP961935
*D.nanchangensis*	HJAUP C1074^T^	OQ942889	OQ942895	OQ944460	OQ944454
***D.nanpingensis***	**HJAUP C2517^T^**	**PQ211096**	**PQ211104**	**PQ303678**	—
*D.narathiwatensis*	MFLUCC 23–0216	OR354400	OR510861	OR481049	OR481046
*D.neorostrata*	MFLUCC 18–0376^T^	MN163008	MN163017	—	—
*D.nonrostrata*	KUNCC 21–10730^T^	OK310699	OK341198	OP413475	OP413481
*D.obclavata*	MFLUCC 18–0329^T^	MN163012	MN163010	—	—
*D.obpyriformis*	MFLUCC 17–1694^T^	—	MG979764	MG988415	MG988422
*D.obpyriformis*	DLUCC 0867	MG979757	MG979765	MG988416	MG988423
*D.pachyconidia*	KUMCC 21–10724^T^	OK310696	OK341194	OP413471	OP413477
*D.palmarum*	MFLUCC 18–1446^T^	MK085062	MK079663	MK087670	MK087660
*D.phangngaensis*	MFLUCC 16–0857^T^	MF077545	MF077556	—	MF135653
*D.phragmiticola*	GUCC 220201^T^	OP749887	OP749880	OP752699	OP749891
*D.phragmiticola*	GUCC 220202^T^	OP749888	OP749881	OP752700	OP749892
*D.rayongensis*	MFLUCC 18–0415^T^	MH457172	MH457137	MH463255	MH463253
*D.rayongensis*	MFLUCC 18–0417	MH457173	MH457138	MH463256	MH463254
*D.rostrata*	MFLUCC 16–0969^T^	MG979758	MG979766	MG988417	MG988424
*D.rostrata*	DLUCC 0885	MG979759	MG979767	—	MG988425
*D.saprophytica*	MFLUCC 18–1238^T^	MW286506	MW287780	MW504069	MW396651
*D.septata*	GZCC 22–0078^T^	ON527939	ON527947	ON533690	ON533683
*D.sinensis*	HJAUP C2044^T^	OP787878	OP787875	—	OP961936
*D.sichuanensis*	KUNCC 23-15519^T^	PP584672	PP584769	—	PP663312
*D.songkhlaensis*	MFLUCC 18–1234^T^	MW286482	MW287755	—	MW396642
*D.suae*	CGMCC 3.24262^T^	OQ874968	OQ732679	OQ870341	OR367670
***D.subtropica***	**HJAUP C2528^T^**	**PQ211099**	**PQ211107**	**PQ303677**	**PQ303684**
***D.subtropica***	**HJAUP C2535**	**PQ211097**	**PQ211105**	—	**PQ303685**
*D.suoluoensis*	MFLUCC 17–0224^T^	MF077546	MF077557	—	MF135654
*D.suoluoensis*	MFLUCC 17–0854	MF077547	MF077558	MZ945510	—
*D.tectonae*	MFLUCC 12–0291^T^	KX751711	KX751713	KX751708	KX751710
*D.tectonae*	MFLU 20–0262	MT232714	MT232719	—	—
*D.tectonigena*	MFLUCC 12–0292^T^	KX751712	KX751714	KX751709	—
*D.thailandica*	MFLUCC 16–0270^T^	MH275060	MH260292	—	MH412767
*D.thysanolaenae*	KUN–HKAS 112710	MW723057	MW879524	—	MW729783
*D.thysanolaenae*	KUN–HKAS 102247^T^	MK045851	MK064091	—	MK086031
*D.tropica*	GZCC 22–0076^T^	ON527935	ON527943	ON533687	ON533679
*D.verrucosa*	GZCC 20–0434^T^	MZ868771	MZ868762	MZ892983	MZ892977
*D.wuzhishanensis*	GZCC 22–0077^T^	ON527938	ON527946	—	ON533682
*D.xinpingensis*	KUNCC 22–12669	OQ874969	OQ732680	—	—
*D.xinpingensis*	KUNCC 22–12667^T^	OQ874970	OQ732681	OQ870340	OR367671
*D.xishuangbannaensis*	KUMCC 17–0290^T^	MH275061	MH260293	MH412754	MH412768
*D.yichunensis*	HJAUP C1065^T^	OQ942885	OQ942892	OQ944459	OQ944453
*D.yongxiuensis*	JAUCC 4725^T^	OK562388	OK562394	—	OK562406
*D.yongxiuensis*	JAUCC 4726^T^	OK562389	OK562395	—	OK562407
*D.yunjushanensis*	JAUCC 4723^T^	OK562392	OK562398	—	OK562410
*D.yunjushanensis*	JAUCC 4724^T^	OK562393	OK562399	—	OK562411
***D.yunjushanensis***	**HJAUP C1307**	**PQ211101**	**PQ211109**	**PQ303675**	**PQ303686**
*D.yunnansis*	MFLUCC 20–0153^T^	MW081541	MW081546	MW151861	MW084995
*Fluminicolasaprophytica*	MFLUCC 15–0976^T^	MF374358	MF374367	MF370954	MF370956
*Myrmecridiumbanksiae*	CPC 19852 = CBS 132536^T^	JX069871	JX069855	—	—
*M.schulzeri*	CBS 100.54	EU041769	EU041826	—	—
*Papulosaamerospora*	AFTOL–ID 748	—	DQ470950	DQ470901	DQ471069
*Pleurophragmiumbambusinum*	MFLUCC 12–0850	KU940161	KU863149	—	KU940213
*Pseudostanjehughesiaaquitropica*	MFLUCC 16–0569^T^	MF077548	MF077559	—	MF135655
*P.lignicola*	MFLUCC 15–0352^T^	MK828643	MK849787	MN124534	MN194047
*Wongiagriffinii*	DAR 80512^T^	KU850473	KU850471	—	—

Notes: The ex-type cultures are indicated using “^T^” after strain numbers; “—” stands for no sequence data in GenBank. **AFTOL**: Assembling the Fungal Tree of Life; **CBS**: Central Bureau voor Schimmel cultures, Utrecht, The Netherlands; **CGMCC**: China General Microbiological Culture Collection Center, Institute of Microbiology, Chinese Academy of Sciences, Beijing, China; **CPC**: Collection of P.W. Crous; **DAR**: Plant Pathology Herbarium, Orange Agriculture Institute, NSW, Australia; **DLUCC**: Dali University Culture Collection, Yunnan; **GZCC**: Guizhou Culture Collection China; **HFJAU**: Herbarium of Fungi, Jiangxi Agricultural University; **HKUCC**: The University of Hong Kong Culture Collection, Hong Kong, China; **JAUCC**: Jiangxi Agricultural University Culture Collection; **KUMCC**: Kunming Institute of Botany Culture Collection; **HKAS**: Kunming Institute of Botany Academia Sinica, Yunnan, China; **MFLU**: the herbarium of Mae Fah Luang University, Chiang Rai, Thailand; **MFLUCC**: Mae Fah Luang University Culture Collection, Chiang Rai, Thailand.

## ﻿Results

### ﻿Molecular phylogeny

**Figure 1.**
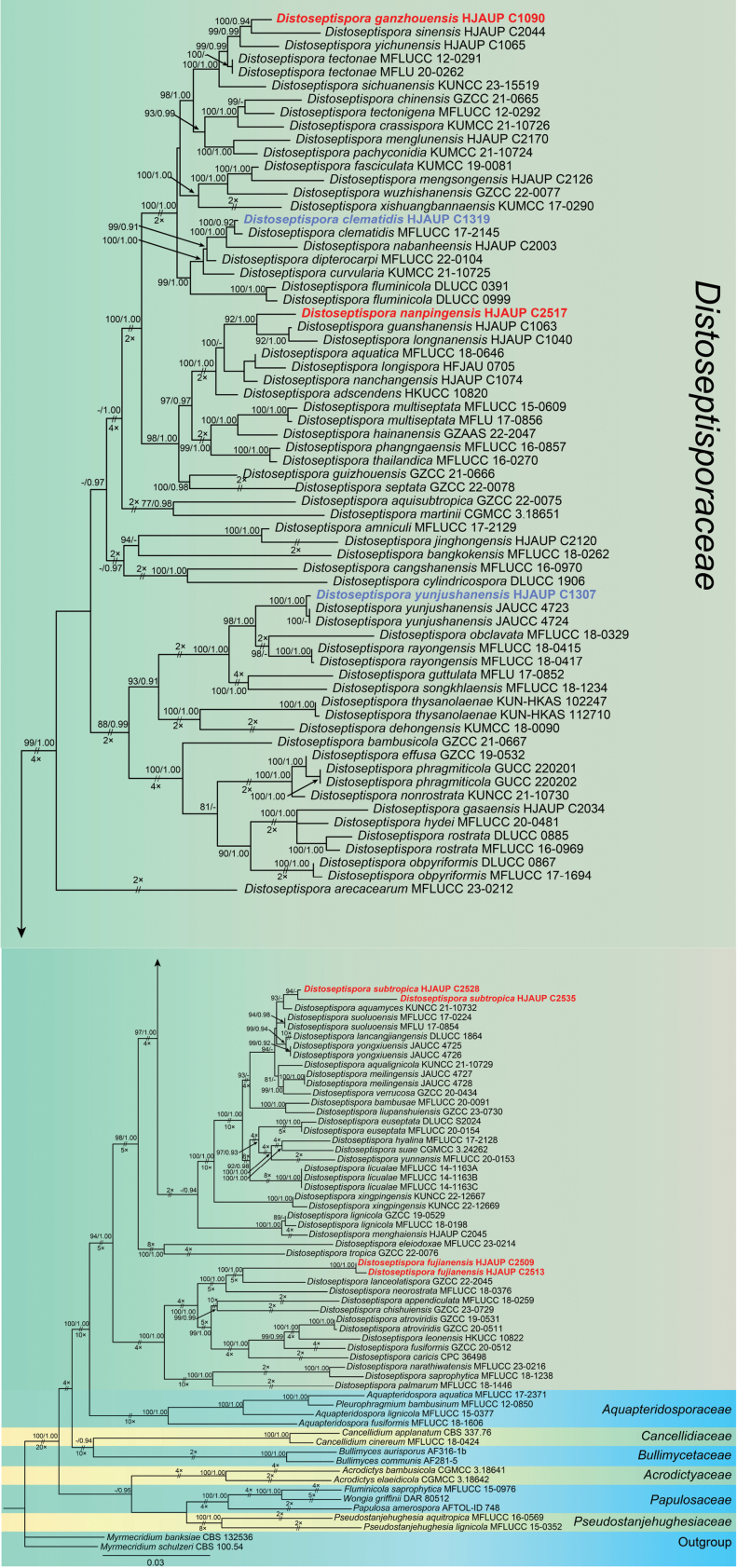
Maximum-likelihood majority rule consensus tree for Distoseptisporaceae and related families using ITS, LSU, *RPB2*, and *TEF1* sequence data. Bootstrap support values for maximum-likelihood greater than 75% and Bayesian posterior probabilities greater than 0.90 are shown near the nodes. The tree is rooted with *Myrmecridiumschulzeri* (CBS 100.54) and *M.banksiae* (CBS 132536). New strains identified in this study are shown in blue; new species are shown in red. Some branches were shortened according to the indicated multipliers, and these are indicated by the symbol (//).

Based on the combined sequences of ITS, LSU, *RPB2* and *TEF1*, eight strains from the present study were analyzed for their phylogenetic relationships within the family Distoseptisporaceae and related families (Acrodictyaceae, Aquapteridosporaceae, Bullimycetaceae, Cancellidiaceae, Papulosaceae, and Pseudostanjehughesiaceae), using 126 strains representing 104 species (Table 1). The combined sequence alignment consisted of 2841 columns (ITS:1–579, LSU:580–1372, *RPB2*:1373–2011, *TEF1*:2012–2841), including 1455 distinct patterns, 1124 parsimony-informative sites, 271 singleton sites, and 1446 constant sites. *Myrmecridiumbanksiae* (CBS 132536) and *M.schulzeri* (CBS 100.54) were used as the outgroup. Maximum-likelihood (ML) and Bayesian inference (BI) analyses of the combined sequences resulted in a phylogenetic reconstruction with essentially similar topological structures. The best-scoring ML tree (lnL = -38006.722) with ultrafast bootstrap values from ML analyses and posterior probabilities from MrBayes analysis are shown in the first and second values above the nodes in Fig. 1. Based on multilocus phylogeny and morphology, the eight strains in this study were assigned to four new species, namely *D.fujianensis*, *D.ganzhouensis*, *D.nanpingensis*, and *D.subtropica*, and two known species, viz. *D.clematidis*, and *D.yunjushanensis*.

### ﻿Taxonomy

#### ﻿ Distoseptispora clematidis

Taxon classificationFungiDistoseptisporalesDistoseptisporaceae

Phukhams., M.V. de Bult & K.D. Hyde, Fungal Diversity 102: 168 (2020)

9B5EA513-0CAE-5B81-B702-D9F4C159893A

Index Fungorum: IF557301

Facesoffungi Number: FoF07261

Fig. 2

##### Description.

***Saprobic*** on dead branches in a terrestrial habitat. **Sexual morph**: Undetermined. **Asexual morph**: Hyphomycetous. ***Colonies*** on natural substrate effuse, scattered, dark brown to black, hairy. ***Mycelium*** partly superficial, partly immersed in the substratum, composed of branched, septate, smooth, pale brown to brown hyphae. ***Conidiophores*** macronematous, mononematous, unbranched, solitary or in groups, erect, straight or slightly flexuous, cylindrical, smooth, brown to dark brown, 2–5-septate, robust at the base, 25–48.5 × 5–8 µm (x̄ = 34.9 × 6.1 µm, n = 12). ***Conidiogenous cells*** monoblastic, integrated, terminal, cylindrical, pale brown to brown, smooth, flat at the conidiogenous loco. ***Conidia*** acrogenous, solitary, dry, obclavate, straight or curved, reddish-brown and slightly paler towards the apex, 26–40-distoseptate, smooth, 150–270 × 11–15 µm (x̄ = 195.1 × 12 µm, n= 30), tapering to 2.5–7.5 µm near the apex, 2.5–5.5 µm wide at the truncate base.

##### Culture characteristics.

***Colonies*** on PDA reaching 76–80 mm diam. after 4 weeks in an incubator under dark conditions at 25 °C, circular, surface velvety, with brown and denser mycelium at the center, becoming black at the margin with an obvious boundary, reverse black and sparser toward the boundary.

**Figure 2.**
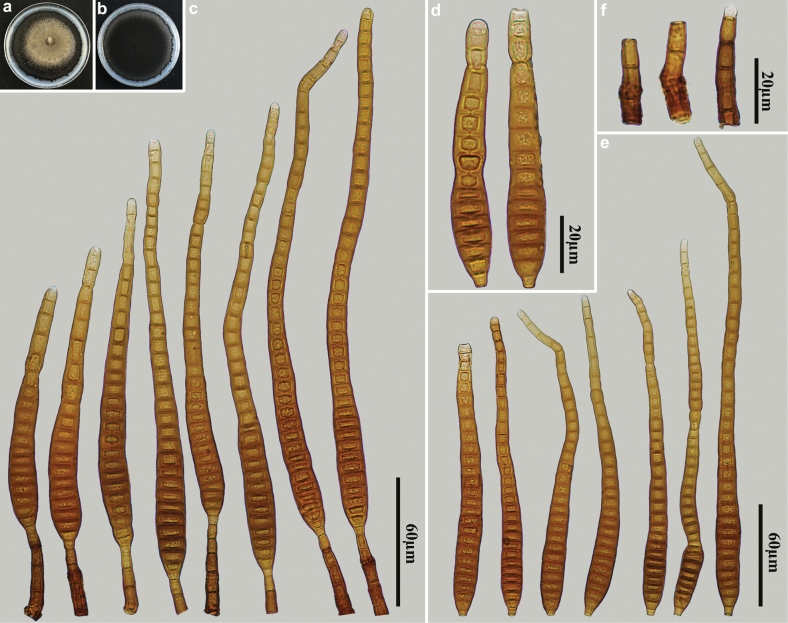
*Distoseptisporaclematidis* (HJAUP M1319) **a** surface of colony after 4 weeks on PDA **b** reverse of colony after 4 weeks on PDA **c** conidiophores, conidiogenous cells and conidia **d, e** conidia **f** conidiophores.

##### Material examined.

China • Jiangxi Province, Ganzhou City, Jiulianshan National Nature Reserve, 24°31'N, 114°27'E, on decaying wood of an unidentified broadleaf tree, 29 June 2022, Y.F. Hu (HJAUP M1319, living culture HJAUP C1319).

##### Notes.

*Distoseptisporaclematidis* was introduced by Phukhamsakda et al. (2020), isolated from the dried stem of *Clematissikkimensis* in Thailand. Phylogenetic analysis shows that our new isolate (HJAUP C1319) clusters with *D.clematidis* Phukhams., M.V. de Bult & K.D. Hyde (MFLUCC 17–2145), with 100% ML/0.92 BI support. Morphologically, our isolate aligns well with the holotype description of *D.clematidis*. Comparisons of their nucleotide sequences showed 4 (0.7%, including one gap), 3 (0.6%, no gaps), and 2 (0.3%, no gaps) nucleotide differences in the ITS, LSU, and *RPB2* regions, respectively, indicating that they are the same species.

#### ﻿ Distoseptispora fujianensis

Taxon classificationFungiDistoseptisporalesDistoseptisporaceae

M.G. Liao & Jian Ma sp. nov.

45A13E5B-5424-50DC-BE6D-6E32F92A5BD4

Index Fungorum: IF902665

Fig. 3

##### Type.

China • Fujian Province, Nanping City, Wuyishan National Nature Reserve, 27°43′N, 117°41′E, on decaying wood of an unidentified broadleaf tree, 16 October 2023, Y.F. Hu (holotype HJAUP M2509; ex-type living culture HJAUP C2509).

##### Etymology.

In reference to Fujian Province, where the fungus was collected.

##### Description.

***Saprobic*** on dead branches in a terrestrial habitat. **Sexual morph**: Undetermined. **Asexual morph**: Hyphomycetous. ***Colonies*** on natural substrate effuse, scattered, dark brown to black, hairy. ***Mycelium*** partly superficial, partly immersed in the substratum, composed of branched, septate, smooth, pale brown to brown hyphae. ***Conidiophores*** macronematous, mononematous, unbranched, solitary or in groups of 2 or 3, straight or slightly flexuous, cylindrical, 6–9-septate, brown to dark brown, 86–127 × 4.5–6 µm (x̄ = 103.4 × 5.2 µm, n = 11). ***Conidiogenous cells*** monoblastic, integrated, terminal, cylindrical, brown, smooth, determinate, flat at the conidiogenous loco. ***Conidia*** solitary, acrogenous, dry, obclavate or ellipsoidal, smooth, 6–8-distoseptate, brown, apical cell paler, 28–47 × 8–14 µm (x̄ = 37.7 × 10.3 µm, n = 23), tapering to 4.5–9 µm near the apex, 4.5–7 µm wide at the truncate base.

**Figure 3.**
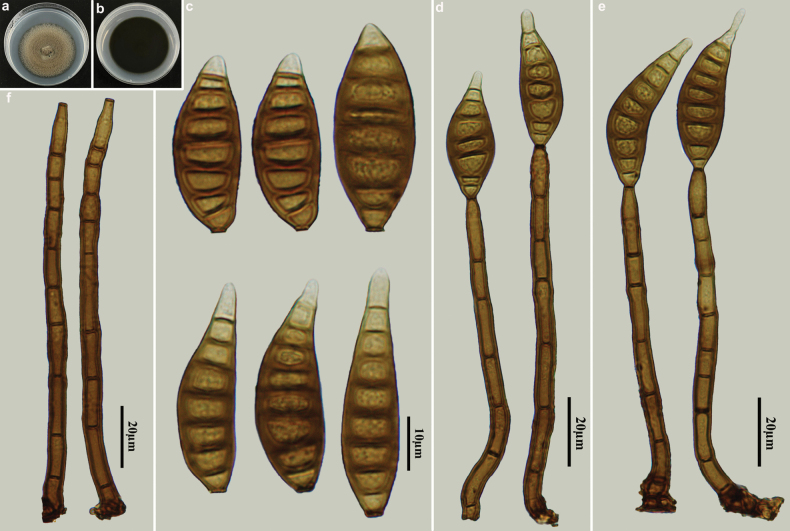
*Distoseptisporafujianensis* (HJAUP M2509, holotype) **a** surface of colony after 4 weeks on PDA **b** reverse of colony after 4 weeks on PDA **c** conidia **d, e** conidiophores, conidiogenous cells and conidia **f** conidiophores.

##### Culture characteristics.

***Colonies*** on PDA reaching 60–63 mm diam. after 4 weeks in an incubator under dark conditions at 25 °C, circular, surface velvety, with pale brown and denser mycelium and the black edge, reverse black.

##### Additional specimen examined.

China • Fujian Province, Nanping City, Wuyishan National Nature Reserve, 27°43′N, 117°41′E, on decaying wood of an unidentified broadleaf tree, 16 October 2023, Y.F. Hu (paratype HJAUP M2513, living culture HJAUP C2513).

##### Notes.

Phylogenetic analyses shows that two strains of *D.fujianensis* (HJAUP C2509 and HJAUP C2513) form a highly support clade, sister to *D.lanceolatispora* X.M. Chen & Y.Z. Lu (GZCC 22-2045), with 100% ML/1.00 BI support. A BLASTn search of GenBank reveals that the sequences of *D.fujianensis* (HJAUP C2509) and *D.lanceolatispora* (GZCC 22-2045) share 96% similarity (504/527, 9 gaps) in ITS, 99% similarity (567/572, 1 gap) in LSU, 92% similarity (990/1075, no gaps) in *RPB2*, and 98% similarity (884/906, no gaps) in *TEF1*. Moreover, *D.fujianensis* differs from *D.lanceolatispora* (Chen et al. 2024) by having shorter conidiophores (86–127 µm vs. 120–190 µm) and smaller conidia (28–47 × 8–14 µm vs. 31–90 × 9.5–15 µm). In addition, *D.fujianensis* further differs from *D.lanceolatispora* by its terrestrial habitat, as opposed to the freshwater habitat of *D.lanceolatispora*.

#### ﻿ Distoseptispora ganzhouensis

Taxon classificationFungiDistoseptisporalesDistoseptisporaceae

M.G. Liao & Jian Ma sp. nov.

AA60154A-A2A3-59FE-BE85-5A83CCC60E19

Index Fungorum: IF902666

Fig. 4

##### Type.

China • Jiangxi Province, Ganzhou City, Jiulianshan National Nature Reserve, 24°31'N, 114°27'E, on decaying wood of an unidentified broadleaf tree, 29 June 2022, Y.F. Hu (holotype HJAUP M1090; ex-type living culture HJAUP C1090).

##### Etymology.

In reference to the locality, Ganzhou city, where the fungus was collected.

##### Description.

***Saprobic*** on dead branches in a terrestrial habitat. **Sexual morph**: Undetermined. **Asexual morph**: Hyphomycetous. ***Colonies*** on natural substratum effuse, hairy, and olivegreen to pale brown. ***Mycelium*** partly superficial, partly immersed in the substratum, composed of branched, septate, smooth, pale brown to brown hyphae. ***Conidiophores*** macronematous, mononematous, solitary, unbranched, straight or flexuous, smooth, 5–10 septate, cylindrical, brown to dark brown, paler towards the apex, determinate or sometimes with a cylindrical, enteroblastic percurrent extension, 54–93 × 4.5–7 µm, (x̄ = 73.1 × 6.2 µm, n = 15). ***Conidiogenous cells*** monoblastic, integrated, terminal, cylindrical, pale brown to brown, smooth, flat at the conidiogenous loco. ***Conidia*** solitary, acrogenous, obclavate, straight or curved, pale brown to brown, 10–23-distoseptate, smooth, 59–139 × 11.5–16.5 µm (x̄ = 96.4 × 13.6 µm, n = 30), tapering to 4–8.5 µm near the apex, 3–6 µm wide at the truncate base.

**Figure 4.**
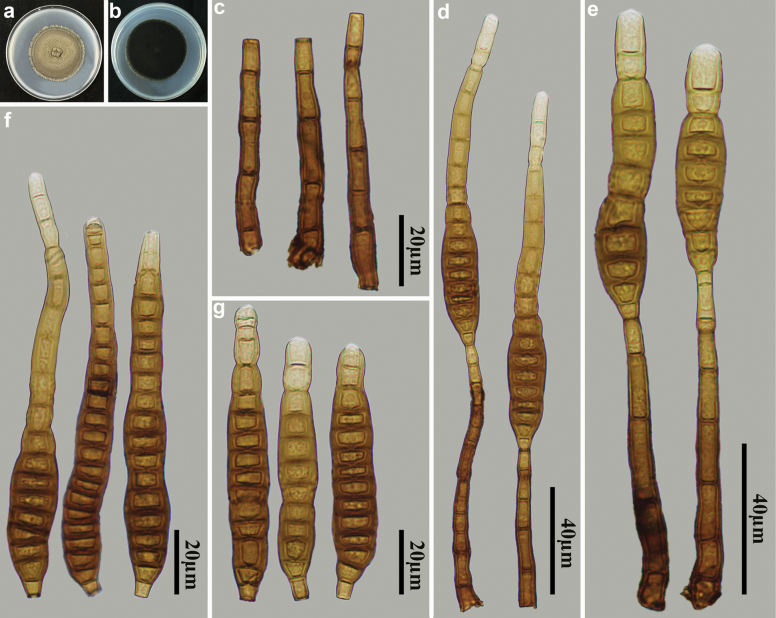
*Distoseptisporaganzhouensis* (HJAUP M1090, holotype) **a** surface of colony after 4 weeks on PDA **b** reverse of colony after 4 weeks on PDA **c** conidiophores **d, e** conidiophores, conidiogenous cells and conidia **f, g** conidia.

##### Culture characteristics.

***Colonies*** on PDA reaching 54 mm diam. after 4 weeks in an incubator under dark conditions at 25 °C, circular, surface velvety, with dense and brown mycelium, dark brown to pale at the margin, reverse dark brown to black.

##### Notes.

Phylogenetic analyses shows that *D.ganzhouensis* (HJAUP C1090) clusters with *D.sinensis* Jing W. Liu, X.G. Zhang & Jian Ma (HJAUP C2044), with 100% ML/0.94 BI support. A BLASTn search of GenBank reveals that the sequences of *D.ganzhouensis* (HJAUP C1090) and *D.sinensis* (HJAUP C2044) share 98% similarity (555/565, one gaps) in ITS, 99% similarity (557/559, two gaps) in LSU, and 99% similarity (925/930, no gaps) in *TEF1*. Moreover, *D.ganzhouensis* is significantly different from *D.sinensis* (Liu et al. 2023) by having longer conidiophores (54–93 µm vs. 23.5–56.5 µm) and wider conidia (11.5–16.5 µm vs. 10–12 µm).

#### ﻿ Distoseptispora nanpingensis

Taxon classificationFungiDistoseptisporalesDistoseptisporaceae

M.G. Liao & Jian Ma sp. nov.

8B879E1C-FA56-505E-975B-0F545593424D

Index Fungorum: IF902669

Fig. 5

##### Type.

China • Fujian Province, Nanping City, Wuyishan National Nature Reserve, 27°43′N, 117°41′E, on decaying wood of an unidentified broadleaf tree, 16 October 2023, Y.F. Hu (holotype HJAUP M2517; ex-type living culture HJAUP C2517).

##### Etymology.

In reference to the locality, Nanping city, where the fungus was collected.

##### Description.

***Saprobic*** on dead branches in a terrestrial habitat. **Sexual morph**: Undetermined. **Asexual morph**: Hyphomycetous. ***Colonies*** on natural substrate effuse, scattered, dark brown to black, hairy. ***Mycelium*** partly superficial, partly immersed in the substratum, composed of branched, septate, smooth, pale brown to brown hyphae. ***Conidiophores*** macronematous, mononematous, unbranched, solitary, straight or slightly flexuous, cylindrical, smooth, 1–3-septate, brown to dark brown, 8.5–28 × 5–7 µm (x̄ = 18.1 × 6.1 µm, n = 10). ***Conidiogenous cells*** monoblastic, integrated, terminal, determinate, cylindrical, flat at the conidiogenous loco. ***Conidia*** solitary, acrogenous, dry, obclavate, straight or curved, sometimes with a swollen cell, reddish-brown and slightly paler towards the apex, 28–41-distoseptate, sometimes constricted at the septa, smooth, sometimes with percurrent regeneration forming a secondary conidium from the conidial apex, 169–282 × 12–17.5 µm (x̄ = 227.1 × 14.8 µm, n = 20), tapering to 5–9 µm near the apex, 3.5–5.5 µm wide at the truncate base.

**Figure 5.**
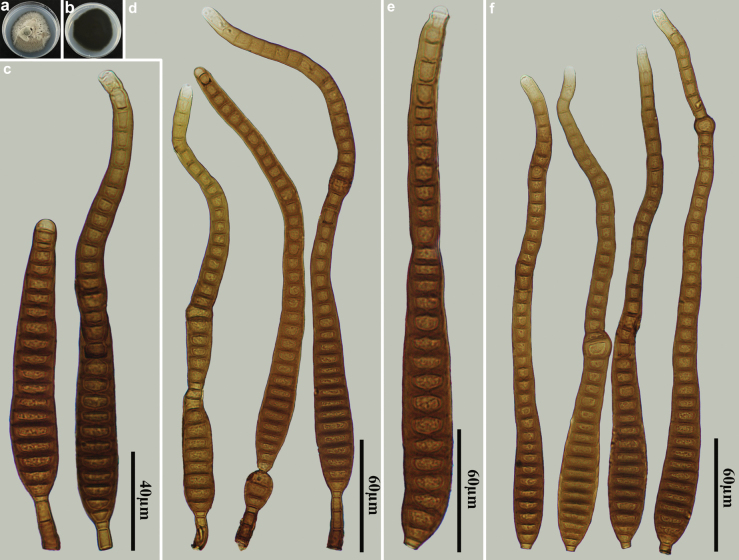
*Distoseptisporananpingensis* (HJAUP M2517, holotype) **a** surface of colony after 4 weeks on PDA **b** reverse of colony after 4 weeks on PDA **c, d** conidiophores, conidiogenous cells and conidia **e, f** Conidia.

##### Culture characteristics.

***Colonies*** on PDA reaching 67–71 mm diam. after 4 weeks in an incubator under dark conditions at 25 °C, irregularly circular, surface velvety with white to pale brown and denser mycelium, becoming black at the margin, reverse black.

##### Notes.

Phylogenetic analyses shows that *D.nanpingensis* (HJAUP C2517) forms a distinct clade, sister to the clade containing *D.guanshanensis* Y. F. Hu & Jian Ma (HJAUP C1063) and *D.longnanensis* Y.F. Hu & Jian Ma (HJAUP C1040), with 92% ML/1.00 BI support. A BLASTn search of GenBank reveals that the sequences of *D.nanpingensis* (HJAUP C2517) and *D.guanshanensis* (HJAUP C1063) share 99% similarity (602/608, no gaps) in ITS, 99% similarity (546/547, no gaps) in LSU, 98% similarity (906/923, four gaps) in *RPB2*, and 98% similarity (906/927, no gaps) in *TEF1*. The sequences of *D.longnanensis* (HJAUP C1040) share 98% similarity (608/621, five gaps) in ITS, 99% similarity (560/565, two gaps) in LSU, and 98% similarity (903/923, no gaps) in *TEF1*. Moreover, *D.nanpingensis* is significantly different from *D.guanshanensis* (Hu et al. 2023) by having shorter conidiophores (8.5–28 µm vs. 15.4–44.7 µm), and longer conidia (169–282 µm vs. 96.5–255.3 μm) sometimes with percurrent regeneration forming a secondary conidium from the conidial apex. *Distoseptisporananpingensis* also differs from *D.longnanensis* (Hu et al. 2023) by having shorter conidiophores (8.5–28 µm vs. 77–171 μm) and larger conidia (169–282 × 12–17.5 µm vs. 54–87 × 8.2–14 μm) with more distosepta (28–41 vs. 4–8).

#### ﻿ Distoseptispora subtropica

Taxon classificationFungiDistoseptisporalesDistoseptisporaceae

M.G. Liao & Jian Ma sp. nov.

45494B08-127D-5C8B-A9F2-A1B712FD672C

Index Fungorum: IF902670

Fig. 6

##### Type.

China • Fujian Province, Nanping City, Wuyishan National Nature Reserve, 27°43′N, 117°41′E, on decaying wood of an unidentified broadleaf tree, 16 October 2023, YF. Hu (holotype HJAUP M2528; ex-type living culture HJAUP C2528).

##### Etymology.

In reference to the subtropical climate in which the species was collected.

##### Description.

***Saprobic*** on dead branches in a terrestrial habitat. **Sexual morph**: Undetermined. **Asexual morph**: Hyphomycetous. ***Colonies*** on natural substrate effuse, scattered, dark brown to black, hairy. ***Mycelium*** partly superficial, partly immersed in the substratum, composed of branched, septate, smooth, pale brown to brown hyphae. ***Conidiophores*** macronematous, mononematous, solitary or in groups, unbranched, erect, straight or slightly flexuous, cylindrical, smooth, 5–10-septate, brown to dark brown and paler towards the apex, 125–262 × 6–10.5 µm (x̄ = 181.7 × 6.7 µm, n = 12). ***Conidiogenous cells*** monoblastic or ployblastic, integrated, terminal, cylindrical, pale brown, smooth, determinate or sometimes with cylindrical, enteroblastic percurrent extensions. ***Conidia*** acropleurogenous, solitary, dry, straight or slightly curved, obclavate, rostrate, verrucose, 5–10-euseptate, pale brown to brown, 64–147 × 12–15 µm (x̄ = 90.1 × 13.3 µm, n = 23), tapering to 2.5–4.5 µm near the apex, 4.5–7 µm wide at the truncate base.

**Figure 6.**
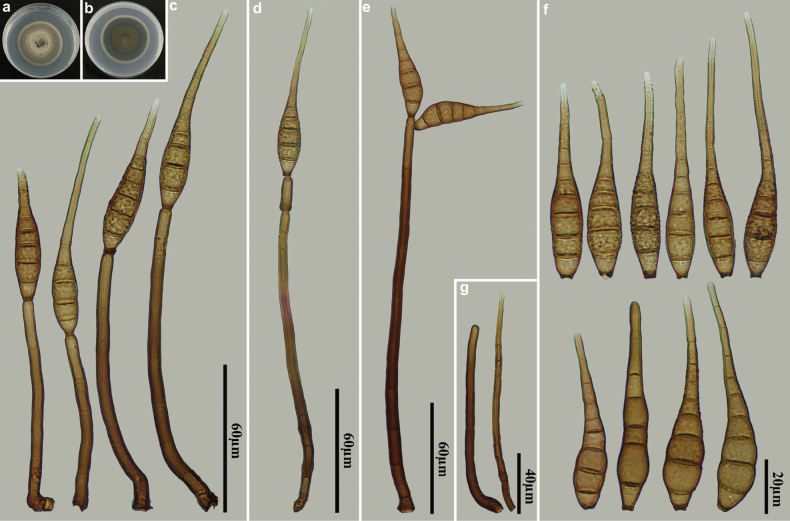
*Distoseptisporasubtropica* (HJAUP M2528, holotype) **a** surface of colony after 4 weeks on PDA **b** reverse of colony after 4 weeks on PDA **c–e** conidiophores, conidiogenous cells and conidia **f** conidia **g** conidiophores.

##### Culture characteristics.

***Colonies*** on PDA reaching 57–58 mm diam. after 4 weeks in an incubator under dark conditions at 25 °C, circular, surface velvety, with yellow to pale brown and denser mycelium at the center, becoming dark brown at the ring with a pale edge, reverse brown at the central parts with a dark brown inner ring and an outer pale brown halo.

##### Additional specimen examined.

China • Fujian Province, Nanping City, Wuyishan National Nature Reserve, 27°43′N, 117°41′E, on decaying wood of an unidentified broadleaf tree, 16 October 2023, Y.F. Hu (paratype HJAUP M2535; living culture HJAUP C2535).

##### Notes.

Strains HJAUP C2528 and HJAUP C2535 grouped together with 94% ML/0.81 BI support. Comparisons of their nucleotide sequences revealed differences of 12 nucleotides (1.9%, including five gaps) in the ITS region, 7 nucleotides (1.2%, including three gaps) in the LSU region, and 13 nucleotides (1.3%, including two gaps) in the *TEF1* region. Based on the criteria established by Jeewon and Hyde (2016), nucleotide differences greater than 1.5% in the ITS region may suggest a new species. However, aside from slight differences in conidial length (64–147 µm vs. 53–86 µm), there were no significant morphological distinctions. Therefore, we propose to identify these two strains as the same new species, *Distoseptisporasubtropica*. Phylogenetic analyses show that *D.subtropica* (HJAUP C2528 and HJAUP C2535) forms a sister clade to *D.aquamyces* R. Zhu & H. Zhang (KUNCC 21–10732), with 93% ML/0.81 BI support. A BLASTn search of GenBank reveals that the sequences of *D.subtropica* (HJAUP C2528) and *D.aquamyces* (KUNCC 21–10732) share 99% similarity (568/575, two gaps) in ITS, 99% similarity (571/574, three gaps) in LSU, 99% similarity (1035/1040, three gaps) in *RPB2*, and 99% similarity (912/919, no gaps) in *TEF1*. Moreover, *D.subtropica* is significantly different from *D.aquamyces* (Zhang et al. 2022) by having larger conidiophores [125–262 × 6–10.5 µm vs. (78–)91–198 × 4–7 µm], and acropleurogenous, obclavate, larger conidia (64–147 × 12–15 µm vs. 30–95 × 7–12 µm).

#### ﻿ Distoseptispora yunjushanensis

Taxon classificationFungiDistoseptisporalesDistoseptisporaceae

Z.J. Zhai & D.M. Hu, MycoKeys 88: 47 (2022)

B2376547-1216-55EE-A731-1A19FB022BDA

842065

Fig. 7

##### Description.

***Saprobic*** on dead branches in a terrestrial habitat. **Sexual morph**: Undetermined. **Asexual morph**: Hyphomycetous. ***Colonies*** on natural substrate effuse, scattered, dark brown to black, hairy. ***Mycelium*** partly superficial, partly immersed in the substratum, composed of branched, septate, smooth, pale brown to brown hyphae. ***Conidiophores*** macronematous, mononematous, solitary or in groups, unbranched, erect, straight or slightly flexuous, cylindrical, smooth, 3–6-septate, pale brown to brown, 125–243 × 7.5–10.5 µm (x̄ = 185 × 9.9 µm, n = 14). ***Conidiogenous cells*** monoblastic, integrated, terminal, determinate or sometimes with one cylindrical percurrent extension, cylindrical, pale brown, smooth. ***Conidia*** acrogenous, solitary, dry, obclavate or ellipsoidal, 5–10-distoseptate, smooth, brown, paler toward the apex, 67.5–96 × 16–22.5 µm (x̄ = 83.2 × 19.4 µm, n = 30), tapering to 2.5–12.5 µm near the apex, 5–7.5 µm wide at the truncate base.

**Figure 7.**
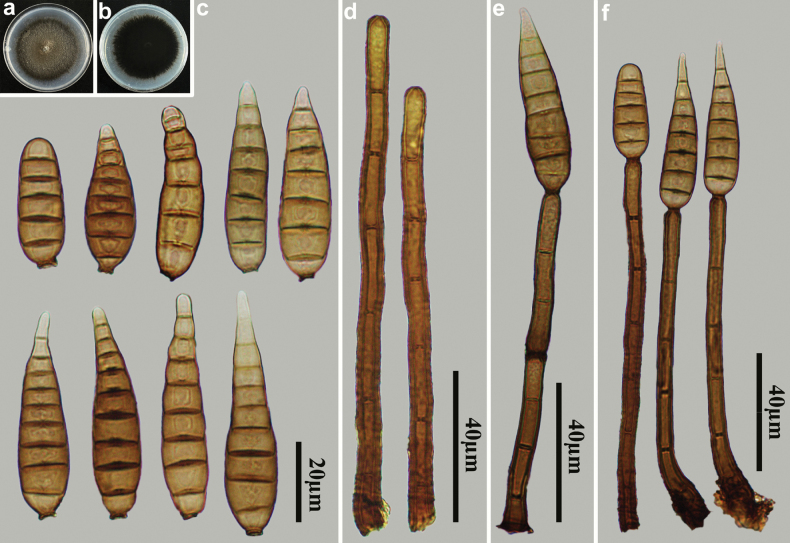
*Distoseptisporayunjushanensis* (HJAUP M1307) **a** surface of colony after 4 weeks on PDA **b** reverse of colony after 4 weeks on PDA **c** conidia **d** conidiophores **e, f** conidiophores, conidiogenous cells and conidia.

##### Culture characteristics.

***Colonies*** on PDA reached 65–75 mm diam. after 4 weeks in an incubator under dark conditions at 25 °C, circular, surface velvety, dark brown at the center, with dense, brown mycelium, black margin entire with flocculous mycelium, reverse black.

##### Material examined.

China • Jiangxi Province, Ganzhou City, Jiulianshan National Nature Reserve, 24°31'N, 114°27'E, on decaying wood of an unidentified broadleaf tree, 29 June 2022, Y.F. Hu (HJAUP M1307, living culture HJAUP C1307).

##### Notes.

*Distoseptisporayunjushanensis*, introduced by Zhai et al. (2022), was originally found on decaying bamboo culms submerged in a freshwater stream in China. Phylogenetic analysis shows that our new isolate (HJAUP C1307) clusters with *D.yunjushanensis* Z. J. Zhai & D. M. Hu (JAUCC 4723 and JAUCC 4724) with 100% ML/1.00 BI support. Comparisons of nucleotide sequences for our new isolate (HJAUP C1307) and *D.yunjushanensis* (JAUCC 4723) showed 7 (1.3%, including three gaps) and 3 (0.5%, including one gap) nucleotide differences in the ITS and LSU regions, respectively. Morphologically, our isolate fits well with the holotype description of *D.yunjushanensis*, except for its longer conidiophores (125–242.5 µm vs. 100–175 µm) and larger conidia [67.5–96 × 16–22.5 µm vs. 39–67.5(–77) × (7–)9.5–13.5(–16.5) μm] with fewer distosepta (4–10 vs. 7–13). In addition, our isolate was collected from a terrestrial habitat, as opposed to the freshwater habitat of the holotype of *D.yunjushanensis*. However, due to the high morphological similarity and only minor molecular differences, our new isolate does not meet the criteria for a new species, and is thus identified as *D.yunjushanensis*.

## ﻿Discussion

*Distoseptispora* is one of the sporidesmium-like genera introduced by Su et al. (2016) based on molecular phylogenetic analyses combined with morphology. It exhibits a conidial ontogeny similar to that of *Sporidesmium* Link and *Ellisembia* Subram., which produce solitary, holoblastic conidia on unbranched conidiophores that may undergo percurrent proliferation. The excessive overlap in morphological features makes it difficult to distinguish *Distoseptispora* from *Sporidesmium* and *Ellisembia* based solely on morphology, however, these genera are not closely related in phylogenetic trees (Su et al. 2016; Yang et al. 2018; Luo et al. 2019). In addition, *Distoseptispora* forms a distinct and well-supported clade sister to *Aquapteridospora* Jiao Yang, K.D. Hyde & Maharachch., which produces 3-euseptate conidia on terminal and intercalary conidiogenous cells with circular scars (Yang et al. 2015; Hyde et al. 2021). Both *Distoseptispora* and *Aquapteridospora* are placed in Distoseptisporales, but are treated as separate families, Distoseptisporaceae K.D. Hyde & McKenzie and Aquapteridosporaceae K.D. Hyde & Hongsanan (Wijayawardene et al. 2020; Hyde et al. 2021).

In recent years, the number of *Distoseptispora* species has steadily increased, now reaching 87 valid taxa, including the four species described in this study. All species have been identified based on morphological and phylogenetic analyses. Liu et al. (2023) listed 63 recognized *Distoseptispora* species with major morphological features, habitat, host, and locality. Subsequently, 24 additional species have been added to the genus, and they are presented in Table 2, following the same format as Liu et al. (2023). *Distoseptispora* species are typically saprobic fungi on dead plant material in freshwater and terrestrial habitats, with the exception of *D.caricis* and *D.palmarum*, which occur on the leaves of *Carex* sp. and the rachis of *Cocosnucifera*, respectively (Crous et al. 2019; Hyde et al. 2019; Hu et al. 2023; Liu et al. 2023; Karimi et al. 2024; Shen et al. 2024). Some *Distoseptispora* species show morphological variation across different specimens, but they can be reliably identified through molecular DNA data. For instance, the conidiophores of *D.tectonae* Doilom & K.D. Hyde are up to 40 × 4–6 μm in the holotype (MFLU 15-3417) (Hyde et al. 2016), but 34–95 × 5–8 μm in a later collection (MFLU 20–0262) (Sun et al. 2020). The conidia of *D.pachyconidia* are 42–136 × 14–22 µm, 8–21-distoseptate, pale-brown with a green tinge in the holotype (HKAS 122179) (Zhang et al. 2022), but 50–242 × 11–20 µm, up to 38-distoseptate, olivaceous to dark brown in another collection (HKAS 123754) (Ma et al. 2022), and (82–)137–246(–296) × (9–)13–16 μm, 14–45-distoseptate, pale brown to brown in yet another collection (HKAS 125824) (Shen et al. 2024). These variations may result from differences in geographic regions, ecological environments, climatic conditions, substrates and habitats, or observation periods. Although some *Distoseptispora* species, such as *D.bambusae*, *D.pachyconidia*, and *D.tectonae*, show morphological variation from the holotype, the BLASTn analysis of multilocus gene sequences shows only minor differences. The use of ITS, LSU, *RPB2* and *TEF1* loci provides a more reliable evaluation of their phylogenetic relationships and taxonomic placements.

**Table 2.** Synopsis of morphological characteristics, habitat, host, and locality compared across *Distoseptispora* species described since Liu et al. (2023).

Species	Conidiophores (μm)	Conida		Habitat	Host/Locality	References
		Size (µm)	Morphology			
*Distoseptisporaarecacearum*	70–140 × 5.1–6.3	25–60 × 7–17	Cylindrical, obclavate to obpyriform or irregular, brown, sometimes with percurrent regeneration forming a secondary conidium from the conidial apex, 4–10-distoseptate	Freshwater	Submerged rachis of *Licualapaludosa*, Thailand	Karimi et al. (2024)
*D.chishuiensis*	80–190 × 4–7	70–130 × 14.5–21	Obpyriform or obclavate, thick-walled, olivaceous or dark brown below, hyaline towards apex, slender and rounded at apex, 13–17-distoseptate	Terrestrial	Dead culms of bamboo, China	Dissanayake et al. (2024)
*D.dipterocarpi*	14–72 × 5–7	31.5–350 × 6.5–12	Cylindrical or obclavate, elongated, olivaceous when young, brown tinge when mature, mostly lighter towards the apex, 10–72-distoseptate	Terrestrial	Woody litter of *Dipterocarpus*, Thailand	Afshari et al. (2023)
*D.eleiodoxae*	71–161 × 5–6.5	31.5–48 × 13.5–15.8	Obpyriform, rostrate, verrucose, brown with dark brown to black cells in the middle, paler towards the apex, 6–7-euseptate	Freshwater	Submerged rachis of *Eleiodoxaconferta*, Thailand	Karimi et al. (2024)
*D.fujianensis*	86–127 × 4.5–6	28–47 × 8–14	obclavate or ellipsoidal, brown, apical cell paler, 6–8-distoseptate	Terrestrial	Unidentified dead branches, China	This study
*D.ganzhouensis*	54–93 × 4.5–7	59–139 × 11.5–16.5	obclavate, pale brown to brown, 10–23-distoseptate	Terrestrial	Unidentified dead branches, China	This study
*D.gasaensis*	104–204 × 4–7.2	44–72 × 6–12	Obclavate or obpyriform, brown to pale brown, rostrate, 5–9-distoseptate	Terrestrial	Unidentified dead branches, China	Hu et al. (2023)
*D.guanshanensis*	15.4–44.7 × 5.2–8.3	96.5–255.3 × 12.3–16.5	Obclavate, pale brown to dark brown, (5–)11–38-distoseptate	Terrestrial	Unidentified dead branches, China	Hu et al. (2023)
*D.hainanensis*	70–130 × 5–8.5	44–117 × 9–18.5	Obclavate or obpyriform, rostrate, brown, verrucose, up to 22-distoseptate	Terrestrial	Unidentified decaying wood, China	Chen et al. (2024)
*D.jinghongensis*	54.6–94.6 × 3.6–4	56.4–127.3 × 7.3–10.9	Obclavate, rostrate, pale brown or brown, 7–17-distoseptate	Terrestrial	Unidentified dead branches, China	Hu et al. (2023)
*D.lanceolatispora*	120–190 × 4–8	31–90 × 9.5–15	Fusiform or lanceolate, rostrate, olivaceous to olivaceous brown, verrucous, with or without apical, hyaline appendages, 5–13-distoseptate	Freshwater	Unidentified submerged wood, China	Chen et al. (2024)
*D.liupanshuiensis*	70–340 × 3.5–7	55–90 × 6–11	Obpyriform, light brown below, hyaline towards apex, 8–10-distoseptate	Terrestrial	Dead culms of bamboo, China	Dissanayake et al. (2024)
*D.longnanensis*	77–171 × 4–8	54–87 × 8.2–14	Obclavate, olivaceous to yellowish-brown or brown, 4–8-septate	Terrestrial	Unidentified dead branches, China	Hu et al. (2023)
*D.menghaiensis*	45.7–82.9 × 3.4–5.1	35.7–48.6 × 7.2–10.9	Obclavate, pale brown to brown, 4–8-distoseptate	Terrestrial	Unidentified dead branches, China	Hu et al. (2023)
*D.menglunensis*	35–52.5 × 6.3–7.5	82.5–172.2 × 12.5–15	Obclavate, brown to dark brown, 14–33-distoseptate	Terrestrial	Unidentified dead branches, China	Hu et al. (2023)
*D.nanpingensis*	8.5–28 × 5–7	169–282 × 12–17.5	obclavate, sometimes with a swollen cell, reddish-brown and slightly paler towards the apex, sometimes with percurrent regeneration forming a secondary conidium from the conidial apex, 28–41-distoseptate	Terrestrial	Unidentified dead branches, China	This study
*D.nanchangensis*	18.2–76.4 × 5.5–8.0	149.1–292.7 × 10.9–17.8	Obclavate, brown to dark brown, sometimes with percurrent regeneration forming a secondary conidium from the conidial apex, apex sometimes with a germ tube or short germination hypha, (17–)21–43-distoseptate	Terrestrial	Unidentified dead branches, China	Hu et al. (2023)
*D.narathiwatensis*	27–155 × 3–6.5(–7)	12–38 × 4.5–8	Solitary or occasionally catenate, subcylindrical to obclavate to conical, olivaceous to brown, apex rounded, often the primary cells of conidia are narrower than the second ones which are often inflated, 1–7-distoseptate	Terrestrial	Dead petiole of *Eugeissonatristis*, Thailand	Karimi et al. (2024)
*D.phragmiticola*	50–145 × 5–8	34–155 × 10.5–17.5	Obclavate, rostrate, pale brown, grayish brown or mid-brown, paler towards the apex, 4–25-septate	Terrestrial	*Phragmitesaustralis*, China	Hyde et al. (2023)
*D.sichuanensis*	15–25 × 4–6	80–145 × 6–17	Obclavate, elongated, yellowish brown to brown, hyaline at the apex, 12–20-distoseptate	Terrestrial	On dead twigs of unidentified host, China	Dissanayake et al. (2024)
*D.suae*	(21–)25–41(–53) × 4–5	(77–)81–101(–109) × 8–10	Obclavate to rostrate, bent at the second or third cell at the base, brown to dark brown, 3–12-euseptate, guttulate, verrucose	Freshwater	On submerged decaying branches, China	Shen et al. (2024)
*D.subtropica*	125–262 × 6–10.5	64–147 × 12–15	acropleurogenous, obclavate, rostrate, verrucose, pale brown to brown, 5–10-euseptate	Terrestrial	Unidentified dead branches, China	This study
*D.xinpingensis*	(97–)105–149(–175) × 4–5	(95–)107–139(–155) × (7–)8–9(–10)	Obclavate, brown, sometimes a second conidium proliferates at the top of the conidia, 8–12-euseptate	Freshwater	On submerged decaying branch, China	Shen et al. (2024)
*D.yichunensis*	17.9–52.7 × 5.3–6.8	113.7–272.7 × 12.2–16.9	Obclavate, pale brown to brown, (14–)22–35-distoseptate	Terrestrial	Unidentified dead branches, China	Hu et al. (2023)

Notes: All conidia are smooth and solitary, except where indicated.

At present, molecular data have greatly advanced the development of fungal taxonomy and phylogenetic relationships, leading to taxonomic revisions and the identification of new species (Naranjo-Ortiz and Gabaldón 2020). While *Distoseptispora* species share morphological similarities within this genus or with other sporidesmium-like taxa, molecular phylogenetic analyses offer strong support for determining their taxonomic status. However, there are no universally accepted criteria for selecting loci for phylogenetic analysis in *Distoseptispora* (Liu et al. 2023), although analyses using ITS, LSU, *RPB2* and *TEF1* loci have yielded more accurate evaluations of phylogenetic relationships and taxonomic placements. Nevertheless, some inconsistencies remain in the morphological characteristics of the same phylogenetic species. For example, the conidiogenous cells of *D.bambusae* are blastic in the holotype (MFLU 20–0261) collected from a terrestrial habitat in China, while they are monoblastic in collections (MFLU 17–1653 and HKAS 125826) from terrestrial and freshwater habitats in Thailand and China, respectively. Additionally, the latter collection (HKAS 125826) shows longer conidiophores and conidia (Sun et al. 2020; Shen et al. 2024). Despite these morphological differences, the strains cluster together with high support in multi-locus phylogenetic analyses using ITS, LSU, *RPB2*, and *TEF1* (Shen et al. 2024). Given this phenomenon, we conclude that certain morphological characters, such as conidiogenesis, traditionally used for species or genus delimitation in sporidesmium-like genera may be insignificant in the current phylogenetic context. As a result, the development of molecular techniques and multi-locus phylogenetic analyses has become a powerful tool for delimiting *Distoseptispora* species in the modern fungal classification. Accordingly, based on previous studies, we proposed four new species and two known species in this study, distinguished by both morphological characteristic and multi-locus (ITS, LSU, *RPB2*, and *TEF1*) phylogenetic analyses.

## Supplementary Material

XML Treatment for Distoseptispora clematidis

XML Treatment for Distoseptispora fujianensis

XML Treatment for Distoseptispora ganzhouensis

XML Treatment for Distoseptispora nanpingensis

XML Treatment for Distoseptispora subtropica

XML Treatment for Distoseptispora yunjushanensis

## ﻿Additional information

### Author ORCIDs

Ming-Gen Liao https://orcid.org/0009-0001-9537-1773

Rafael F. Castañeda-Ruíz https://orcid.org/0000-0003-0063-3265

Jian Ma https://orcid.org/0000-0001-9783-1860

### Data availability

All of the data that support the findings of this study are available in the main text or Supplementary Information.

## Supplementary materials

Supplementary material 1 The concatenated LSU, ITS, *RPB2* and *TEF1* sequences Data type fas https://binary.pensoft.net/file/1234863This dataset is made available under the Open Database License (http://opendatacommons.org/licenses/odbl/1.0/). The Open Database License (ODbL) is a license agreement intended to allow users to freely share, modify, and use this Dataset while maintaining this same freedom for others, provided that the original source and author(s) are credited.Ming-Gen Liao, Xing-Xing Luo, Ya-Fen Hu, Rafael F. Castañeda-Ruíz, Zhao-Huan Xu, Jian Ma

## References

[B1] Afshari N Gomes de Farias AR Bhunjun CS Phukhamsakda C Hyde KD Lumyong S (2023) *Distoseptisporadipterocarpi* sp. nov. (Distoseptisporaceae), a lignicolous fungus on decaying wood of *Dipterocarpus* in Thailand. Current Research in Environmental & Applied Mycology 13(1): 68–78. 10.5943/cream/13/1/5

[B2] Chen XM Tang X Ma J Liu NG Tibpromma S Karunarathna SC Xiao YP Lu YZ (2024) Identification of two new species and a new host record of *Distoseptispora* (Distoseptisporaceae, Distoseptisporales, Sordariomycetes) from terrestrial and freshwater habitats in southern China. MycoKeys 102: 83–105. 10.3897/mycokeys.102.115452 38370857 PMC10873807

[B3] Crous PW Wingfield MJ Lombard L Roets F Swart WJ Alvarado P Carnegie AJ Moreno G Luangsaard J Thangavel R Alexandrova AV Baseia IG Bellanger JM Bessette AE Bessette AR De la Peña-Lastra S García D Gené J Pham THG Heykoop M Malysheva E Malysheva V Martín MP Morozova OV Noisripoom W Overton BE Rea AE Sewall BJ Smith ME Smyth CW Tasanathai K Visagie CM Adamčík S Alves A Andrade JP Aninat MJ Araújo RVB Bordallo JJ Boufleur T Baroncelli R Barreto RW Bolin J Cabero J Caboň M Cafà G Caffot MLH Cai L Carlavilla JR Chávez R de Castro RRL Delgat L Deschuyteneer D Dios MM Domínguez LS Evans HC Eyssartier G Ferreira BW Figueiredo CN Liu F Fournier J Galli-Terasawa LV Gil-Durán C Glienke C Gonçalves MFM Gryta H Guarro J Himaman W Hywel-Jones N Iturrieta-González I Ivanushkina NE Jargeat P Khalid AN Khan J Kiran M Kiss L Kochkina GA Kolařík M Kubátová A Lodge DJ Loizides M Luque D Manjón JL Marbach PAS Massola Jr NS Mata M Miller AN Mongkolsamrit S Moreau PA Morte A Mujic A Navarro-Ródenas A Németh MZ Nóbrega TF Nováková A Olariaga I Ozerskaya SM Palma MA (2019) Fungal Planet description sheets: 951–1041. Persoonia 43(1): 223–425. 10.3767/persoonia.2019.43.06 32214501 PMC7085856

[B4] Dissanayake LS Samarakoon MC Maharachchikumbura SSN Hyde KD Tang X Li QR Mortimer PE Faraj TK Xu JC Kang JC Wanasinghe DN (2024) Exploring the taxonomy and phylogeny of Sordariomycetes taxa emphasizing Xylariomycetidae in Southwestern China. Mycosphere: Journal of Fungal Biology 15(1): 1675–1793. 10.5943/mycosphere/15/1/15

[B5] Dong W Hyde KD Jeewon R Doilom M Yu XD Wang GN Liu NG Hu DM Nalumpang S Zhang H (2021) Towards a natural classification of annulatascaceae-like taxa II: Introducing five new genera and eighteen new species from freshwater. Mycosphere: Journal of Fungal Biology 12(1): 1–88. 10.5943/mycosphere/12/1/1

[B6] Goh TK (1999) Single-spore isolation using a hand-made glass needle. Fungal Diversity 2: 47–63.

[B7] Hu YF Liu JW Luo XX Xu ZH Xia JW Zhang XG Castañeda-Ruíz RF Ma J (2023) Multi-locus phylogenetic analyses reveal eight novel species of *Distoseptispora* from southern China. Microbiology Spectrum 11(6): e02468–e23. 10.1128/spectrum.02468-23 PMC10715003 37905843

[B8] Hyde KD Hongsanan S Jeewon R Bhat DJ McKenzie EHC (2016) Fungal diversity notes 367–490: Taxonomic and phylogenetic contributions to fungal taxa. Fungal Diversity 80: 1–270. 10.1007/s13225-016-0373-x

[B9] Hyde KD Tennakoon DS Jeewon R Bhat DJ Maharachchikumbura SSN Rossi W Leonardi M Lee HB Mun HY Houbraken J Nguyen TTT Jeon SJ Frisvad JC Wanasinghe DN Lücking R Aptroot A Cáceres MES Karunarathna SC Hongsanan S Phookamsak R de Silva NI Thambugala KM Jayawardena RS Senanayake IC Boonmee S Chen J Luo ZL Phukhamsakda C Pereira OL Abreu VP Rosado AWC Bart B Randrianjohany E Hofstetter V Gibertoni TB Soares AM da S Plautz Jr HL Sotão HMP Xavier WKS Bezerra JDP de Oliveira TGL de Souza-Motta CM Magalhães OMC Bundhun D Harishchandra D Manawasinghe IS Dong W Zhang SN Bao DF Samarakoon MC Pem D Karunarathna A Lin CG Yang J Perera RH Kumar V Huang SK Dayarathne MC Ekanayaka AH Jayasiri SC Xiao Y Konta S Niskanen T Liimatainen K Dai YC Ji XH Tian XM Mešić A Singh SK Phutthacharoen K Cai L Sorvongxay T Thiyagaraja V Norphanphoun C Chaiwan N Lu YZ Jiang HB Zhang JF Abeywickrama PD Aluthmuhandiram JVS Brahmanage RS Zeng M Chethana T Wei D Réblová M Fournier J Nekvindová J do Nascimento Barbosa R dos Santos JEF de Oliveira NT Li G-J Ertz D Shang Q-J Phillips AJL Kuo C-H Camporesi E Bulgakov TS Lumyong S Jones EBG Chomnunti P Gentekaki E Bungartz F Zeng X-Y Fryar S Tkalčec Z Liang J Li G Wen T-C Singh PN Gafforov Y Promputtha I Yasanthika E Goonasekara ID Zhao R-L Zhao Q Kirk PM Liu J-K Yan JY Mortimer PE Xu J Doilom M (2019) Fungal diversity notes 1036–1150: Taxonomic and phylogenetic contributions on genera and species of fungal taxa. Fungal Diversity 96(1): 1–242. 10.1007/s13225-019-00429-2

[B10] Hyde KD Bao DF Hongsanan S Chethana K Yang J Suwannarach N (2021) Evolution of freshwater Diaporthomycetidae (Sordariomycetes) provides evidence for five new orders and six new families. Fungal Diversity 107(1): 71–105. 10.1007/s13225-021-00469-7

[B11] Hyde KD Norphanphoun C Ma J Yang HD Zhang JY Du TY Gao Y Gomes de Farias AR He SC He YK Li CJY Li JY Liu XF Lu L Su HL Tang X Tian XG Wang SY Wei DP Xu RF Xu RJ Yang YY Zhang F Zhang Q Bahkali AH Boonmee S Chethana KWT Jayawardena RS Lu YZ Karunarathna SC Tibpromma S Wang Y Zhao Q (2023) Mycosphere notes 387–412 – novel species of fungal taxa from around the world. Mycosphere: Journal of Fungal Biology 14(1): 663–744. 10.5943/mycosphere/14/1/8

[B12] Index Fungorum (2024) Index Fungorum. http://www.indexfungorum.org/Names/Names.asp

[B13] Jeewon R Hyde KD (2016) Establishing species boundaries and new taxa among fungi: Recommendations to resolve taxonomic ambiguities. Mycosphere: Journal of Fungal Biology 7(11): 1669–1677. 10.5943/mycosphere/7/11/4

[B14] Kalyaanamoorthy S Minh BQ Wong TKF von Haeseler A Jermiin LS (2017) ModelFinder: Fast model selection for accurate phylogenetic estimates. Nature Methods 14(6): 587–589. 10.1038/nmeth.4285 28481363 PMC5453245

[B15] Karimi O Chethana KWT de Farias ARG Asghari R Kaewchai S Hyde KD Li QR (2024) Morphology and multigene phylogeny reveal three new species of *Distoseptispora* (Distoseptisporales, Distoseptisporaceae) on palms (Arecaceae) from peatswamp areas in southern Thailand. MycoKeys 102: 55–81. 10.3897/mycokeys.102.112815 38370856 PMC10873808

[B16] Katoh K Standley DM (2013) MAFFT multiple sequence alignment software version 7: Improvements in performance and usability. Molecular Biology and Evolution 30(4): 772–780. 10.1093/molbev/mst010 23329690 PMC3603318

[B17] Konta S Tibpromma S Karunarathna SC Samarakoon MC Steven LS Mapook A Boonmee S Senwanna C Balasuriya A Eungwanichayapant PD Hyde KD (2023) Morphology and multigene phylogeny reveal ten novel taxa in Ascomycota from terrestrial palm substrates (Arecaceae) in Thailand. Mycosphere: Journal of Fungal Biology 14(1): 107–152. 10.5943/mycosphere/14/1/2

[B18] Liu YJ Whelen S Hall BD (1999) Phylogenetic relationships among ascomycetes: Evidence from an RNA polymerse II subunit. Molecular Biology and Evolution 16(12): 1799–1808. 10.1093/oxfordjournals.molbev.a026092 10605121

[B19] Liu JW Hu YF Luo XX Xu ZH Castañeda-Ruíz RF Xia JW Zhang XG Zhang LH Cui RQ Ma J (2023) Morphological and phylogenetic analyses reveal three new species of *Distoseptispora* (Distoseptisporaceae, Distoseptisporales) from Yunnan, China. Journal of Fungi (Basel, Switzerland) 9(4): 470. 10.3390/jof9040470 37108924 PMC10142134

[B20] Luo ZL Hyde KD Liu JK Bhat DJ Bao DF Li WL Su HY (2018) Lignicolous freshwater fungi from China II: Novel *Distoseptispora* (Distoseptisporaceae) species from northwestern Yunnan Province and a suggested unified method for studying lignicolous freshwater fungi. Mycosphere: Journal of Fungal Biology 9(3): 444–461. 10.5943/mycosphere/9/3/2

[B21] Luo ZL Hyde KD Liu JK Maharachchikumbura SSN Jeewon R Bao DF Bhat DJ Lin CG Li WL Yang J Liu NG Lu YZ Jayawardena RS Li JF Su HY (2019) Freshwater Sordariomycetes. Fungal Diversity 99(1): 451–660. 10.1007/s13225-019-00438-1

[B22] Ma J Wang Y Ma LG Zhang YD Castañeda-Ruíz RF Zhang XG (2011) Three new species of *Neosporidesmium* from Hainan, China. Mycological Progress 10(2): 157–162. 10.1007/s11557-010-0685-2

[B23] Ma J Zhang JY Xiao XJ Xiao YP Tang X Boonmee S Kang JC Lu YZ (2022) Multi-gene phylogenetic analyses revealed five new species and two new records of Distoseptisporales from China. Journal of Fungi (Basel, Switzerland) 8(11): 1202. 10.3390/jof8111202 36422023 PMC9697283

[B24] Minh BQ Nguyen MAT von Haeseler A (2013) Ultrafast approximation for phylogenetic bootstrap. Molecular Biology and Evolution 30(5): 1188–1195. 10.1093/molbev/mst024 23418397 PMC3670741

[B25] Naranjo-Ortiz MA Gabaldón T (2020) Fungal evolution: Cellular, genomic and metabolic complexity. Biological Reviews of the Cambridge Philosophical Society 95(5): 1198–1232. 10.1111/brv.12605 32301582 PMC7539958

[B26] Nguyen LT Schmidt HA von Haeseler A Minh BQ (2015) IQ-TREE: A fast and effective stochastic algorithm for estimating maximum-likelihood phylogenies. Molecular Biology and Evolution 32(1): 268–274. 10.1093/molbev/msu300 25371430 PMC4271533

[B27] Phukhamsakda C McKenzie EHC Phillips AJL Jones EBG Bhat DJ Marc S Bhunjun CS Wanasinghe DN Thongbai B Camporesi E Ertz D Jayawardena RS Perera RH Ekanayake AHE Tibpromma S Doilom M Xu J Hyde KD (2020) Microfungi associated with *Clematis* (Ranunculaceae) with an integrated approach to delimiting species boundaries. Fungal Diversity 102(1): 1–203. 10.1007/s13225-020-00448-4

[B28] Rehner SA Buckley E (2005) A beauveria phylogeny inferred from nuclear ITS and EF1-α sequences: Evidence for cryptic diversification and links to Cordyceps teleomorphs. Mycologia 97(1): 84–98. 10.3852/mycologia.97.1.84 16389960

[B29] Ronquist F Teslenko M van der Mark P Ayres DL Darling A Höhna S Larget B Liu L Suchard MA Huelsenbeck JP (2012) MrBayes 3.2: Efficient bayesian phylogenetic inference and model choice across a large model space. Systematic Biology 61(3): 539–542. 10.1093/sysbio/sys029 22357727 PMC3329765

[B30] Shen HW Bao DF Boonmee S Lu YZ Su XJ Li YX Luo ZL (2024) Diversity of *Distoseptispora* (Distoseptisporaceae) taxa on submerged decaying wood from the Red River in Yunnan, China. MycoKeys 102: 1–28. 10.3897/mycokeys.102.116096 38356851 PMC10862349

[B31] Su HY Hyde KD Maharachchikumbura SSN Ariyawansa HA Luo ZL Promputtha I Tian Q Lin CG Shang QJ Zhao YC Chai HM Liu XY Bahkali AH Bhat JD McKenzie EHC Zhou DQ (2016) The families Distoseptisporaceae fam. nov., Kirschsteiniotheliaceae, Sporormiaceae and Torulaceae, with new species from freshwater in Yunnan Province, China. Fungal Diversity 80(1): 375–409. 10.1007/s13225-016-0362-0

[B32] Sun Y Goonasekara ID Thambugala KM Jayawardena RS Wang Y Hyde KD (2020) *Distoseptisporabambusae* sp. nov. (Distoseptisporaceae) on bamboo from China and Thailand. Biodiversity Data Journal 8: e53678. 10.3897/BDJ.8.e53678 PMC7280319 32547305

[B33] Sung GH Sung JM Hywel-Jones NL Spatafora JW (2007) A multi-gene phylogeny of Clavicipitaceae (Ascomycota, fungi): Identification of localized incongruence using a combinational bootstrap approach. Molecular Phylogenetics and Evolution 44(3): 1204–1223. 10.1016/j.ympev.2007.03.011 17555990

[B34] White TJ Bruns TD Lee SB Taylor JW (1990) Amplification and direct sequencing of fungal ribosomal RNA genes for phylogenetics. In PCR Protocols: A guide to methods and applications 18: 315–322. 10.1016/B978-0-12-372180-8.50042-1

[B35] Wijayawardene NN Hyde KD Al-Ani LK Tedersoo L Haelewaters D Rajeshkumar KC Zhao R Aptroot A Leontyev DV Saxena RK Tokarev YS Dai D Letcher PM Stephenson SL Ertz D Lumbsch HT Kukwa M Issi IV Madrid H Phillips AJ Selbmann L Pfliegler WP Horváth E Bensch K Kirk PM Kolaříková K Raja HA Radek R Papp V Dima VS Ma J Malosso E Takamatsu S Rambold G Gannibal PB Triebel D Gautam AK Avasthi S Suetrong S Timdal E Fryar SC Delgado G Réblová M Doilom M Dolatabadi S Pawłowska J Humber RA Kodsueb R Sánchez-Castro I Goto BT Silva DK Souza FA Oehl F Silva GA Błaszkowski J Jobim K Maia LC Barbosa FR Fiuza PO Divakar PK Shenoy BD Castañeda-Ruiz RF Somrithipol S Lateef AA Karunarathna SC Tibpromma S Mortimer PE Wanasinghe DN Phookamsak R Xu J Wang Y Tian F Alvarado P Li DW Kušan I Matočec N Mešić A Tkalčec Z Maharachchikumbura SS Papizadeh M Heredia G Wartchow F Bakhshi M Boehm EW Youssef NH Hustad VP Lawrey JD Santiago AE Bezerra JD Souza-Motta CM Firmino AL Tian Q Houbraken J Hongsanan S Tanaka K Dissanayake AJ Monteiro JS Grossart H Suija A Weerakoon G Etayo J Tsurykau A Vazquez V Mungai PG Damm U Li Q Zhang H Boonmee S Lu Y Becerra AG Kendrick BJ Brearley FQ Motiejūnaitė J Sharma BR Khare R Gaikwad SB Wijesundara D Tang L He M Flakus A Rodriguez-Flakus P Zhurbenko MP McKenzie EH Stadler M Bhat DJ Liu JK Raza M Jeewon R Nassonova ES Prieto M Jayalal RU Erdoğdu M Yurkov AM Schnittler M Shchepin ON Novozhilov YK Silva-Filho AG Gentekaki E Liu P Cavender JC Kang Y Mohammad SN Zhang L Xu R Li YM Dayarathne MC Ekanayaka AH Wen T Deng C Pereira OL Navathe S Hawksworth DL Fan XL Dissanayake LS Kuhnert E Thines M (2020) Outline of fungi and fungus-like taxa. Mycosphere: Journal of Fungal Biology 11(1): 1060–1456. 10.5943/mycosphere/11/1/8

[B36] Xia JW Ma YR Li Z Zhang XG (2017) Acrodictys-like wood decay fungi from southern China, with two new families Acrodictyaceae and Junewangiaceae. Scientific Reports 7(1): 7888. 10.1038/s41598-017-08318-x 28801663 PMC5554248

[B37] Yang J Maharachchikumbura SSN Hyde KD Bhat DJ McKenzie EHC Bahkali AH Gareth Jones EBG Liu ZY (2015) *Aquapteridosporalignicola* gen. et sp. nov., a new hyphomycetous taxon (Sordariomycetes) from wood submerged in a freshwater stream. Cryptogamie. Mycologie 36(4): 469–478. 10.7872/crym/v36.iss4.2015.469

[B38] Yang J Maharachchikumbura SSN Liu JK Hyde KD Jones EBG Al-Sadi AM Liu ZY (2018) *Pseudostanjehughesiaaquitropica* gen. et sp. nov. and *Sporidesmium**sensu lato* species from freshwater habitats. Mycological Progress 17(5): 591–616. 10.1007/s11557-017-1339-4

[B39] Yang J Liu LL Jones EBG Li WL Hyde KD Liu ZY (2021) Morphological variety in *Distoseptispora* and introduction of six novel species. Journal of Fungi (Basel, Switzerland) 7(11): 945. 10.3390/jof7110945 34829232 PMC8620209

[B40] Zhai ZJ Yan JQ Li WW Gao Y Hu HJ Zhou JP Song HY Hu DM (2022) Three novel species of *Distoseptispora* (Distoseptisporaceae) isolated from bamboo in Jiangxi Province, China. MycoKeys 88: 35–54. 10.3897/mycokeys.88.79346 36478919 PMC9633981

[B41] Zhang D Gao FL Jakovlić I Zou H Zhang J Li WX Wang GT (2020) PhyloSuite: An integrated and scalable desktop platform for streamlined molecular sequence data management and evolutionary phylogenetics studies. Molecular Ecology Resources 20(1): 348–355. 10.1111/1755-0998.13096 31599058

[B42] Zhang H Zhu R Qing Y Yang H Li C Wang G Zhang D Ning P (2022) Polyphasic identification of *Distoseptispora* with six new species from fresh water. Journal of Fungi (Basel, Switzerland) 8(10): 1063. 10.3390/jof8101063 36294625 PMC9605234

[B43] Zhao N Luo ZL Hyde KD Su HY Bhat DJ Liu JK Bao DF Hao YE (2018) *Helminthosporiumsubmersum* sp. nov. (Massarinaceae) from submerged wood in north-western Yunnan Province, China. Phytotaxa 348(4): 269–278. 10.11646/phytotaxa.348.4.3

